# Supplementary material for: Characterization, Recombinant Production, and Bioactivity of a Novel Immunomodulatory Protein from Hypsizygus marmoreus
Source: Molecules. 2023 Jun 15;28(12):4796. doi: 10.3390/molecules28124796 (PMC10304396; doi:10.3390/molecules28124796)
Supplement: Supplementary file 1 [file molecules-28-04796-s001.zip › molecules-2342320-supplementary.pdf]

Supplementary Information

Table S1. Physical and chemical properties of immunomodulatory proteins.

**Protein name**	**FIP-hma**
**Number of amino acids**	145
**Molecular weight (kDa)**	14.79
**Theoretical pI**	7.69
**Instability index**	22.85
**Aliphatic index**	90.34
**Grand average of hydropathy**	0.397

Table S2. Summary Table of the Reported Fungal Immunomodulatory Proteins (FIPs)

species	FIPs	accession No.	ref.
Ganoderma australe	c13717	-	[1]
Volvariella volvacea	FIP-vvo	-	[2]
Volvariella volvacea	FIP-vvo82	-	[3]
Volvariella volvacea	FIP-vvo77	-	[3]
Volvariella volvacea	FIP-vvo79	-	[3]
Volvariella volvacea	FIP-vvo80	-	[3]
Volvariella volvacea	FIP-vvo98	-	[3]
Volvariella volvacea	FIP-vvo78	-	[3]
Ganoderma sinense	FIP-gsi	AY449805.1	[4]
Ganoderma tsugae	FIP-gts	-	[5]
Ganoderma lucidum	FIP-glu(LZ-8)	P14945.2	[6]

Trametes versicolor	YZP	AGH06133.1	[7]
Antrodia camphorate	ACA	AAT11911.1	[8]
Ganoderma japonicum	FIP-gja	AAX98241	-
Ganoderma atrum	FIP-gat	AJD79556.1	[9]
Ganoderma microsporum	FIP-gmi	3KCW	[10]
Ganoderma applanatum	FIP-gap1	AEP68179.1	[11]
Ganoderma applanatum	FIP-gap2	ART88472.1	[11]
Chroogomphus rutilus	FIP-cru	AKU37620.1	[12]
Flammulina velutipes	FIP-fve	P80412.1	[13]
Botryobasidium botryosum	FIP-bbo	KDQ10166.1	[14]
Postia placenta	FIP-ppl	AJL35148.1	[15]
Dichomitus squalens	FIP-dsq2	XP_007363541	[16]

Reference

1. González Muñoz A, Botero Orozco KJ, López Gartner GA. Finding of a novel fungal immunomodulatory protein coding sequence in *Ganoderma australe*. *Revista Colombiana Biotecnología*. (2014) 16:90-5.
2. Hsu HC, Hsu CI, Lin RH, Kao CL, Lin JY. Fip-vvo, a new fungal immunomodulatory protein isolated from *Volvariella volvacea*. *Biochemical Journal*. (1997) 323:557-65.
3. Wang Y, Wang Y, Gao YN, Li Y, Wan JN, Yang RH, et al. Discovery and characterization of the highly active fungal immunomodulatory protein fip-vvo82. *Journal of Chemical Information and Modeling*. (2016) 56:2103-14. doi: 10.1021/acs.jcim.6b00087.
4. Li QZ, Wang XF, Chen YY, Lin J, Zhou XW. Cytokines expression induced by *Ganoderma sinensis* fungal immunomodulatory proteins (fip-gsi) in mouse spleen cells. *Applied Biochemistry and Biotechnology*. (2010) 162:1403-13. doi: 10.1007/s12010-010-8916-1.
5. Lin WH, Hung CH, Hsu CI, Lin JY. Dimerization of the N-terminal amphipathic α-helix domain of the fungal immunomodulatory protein from *Ganoderma tsugae* (fip-gts) defined by a yeast two-hybrid system and site-directed mutagenesis. *The journal of biological chemistry*. (1997) 272:20044–8.
6. Kino K, Yamashita A, Yamaoka K, Watanabe J, Tanaka S, Ko K, et al. Isolation and characterization of a new immunomodulatory protein, ling zhi-8 (LZ-8), from *Ganoderma lucidium*.

Journal of Biological Chemistry. (1989) 264:472-8. doi: 10.1016/s0021-9258(17)31282-6.
7. Kuan YC, Wu YJ, Hung CL, Sheu F. *Trametes versicolor* protein YZP activates regulatory B lymphocytes - gene identification through de novo assembly and function analysis in a murine acute colitis model. *Plos One*. (2013) 8:e72422. doi: 10.1371/journal.pone.0072422.
8. Sheu F, Chien PJ, Hsieh KY, Chin KL, Huang WT, Tsao CY, et al. Purification, cloning, and functional characterization of a novel immunomodulatory protein from *Antrodia camphorata* (bitter mushroom) that exhibits TLR2-dependent NF-KB activation and M1 polarization within murine macrophages. *J Agric Food Chem*. (2009) 57:4130-41. doi: 10.1021/jf900469a.
9. Xu H, Kong YY, Chen X, Guo MY, Bai XH, Lu YJ, et al. Recombinant fip-gat, a fungal immunomodulatory protein from *Ganoderma atrum*, induces growth inhibition and cell death in breast cancer cells. *J Agric Food Chem*. (2016) 64:2690-8. doi: 10.1021/acs.jafc.6b00539.
10. Lin CH, Sheu GT, Lin YW, Yeh CS, Huang YH, Lai YC, et al. A new immunomodulatory protein from *Ganoderma microsporum* inhibits epidermal growth factor mediated migration and invasion in A549 lung cancer cells. *Process Biochemistry*. (2010) 45:1537-42. doi: 10.1016/j.procbio.2010.06.006.
11. Zhou S, Guan S, Duan Z, Han X, Zhang X, Fan W, et al. Molecular cloning, codon-optimized gene expression, and bioactivity assessment of two novel fungal immunomodulatory proteins from *Ganoderma applanatum* in *Pichia*. *Appl Microbiol Biotechnol*. (2018) 102:5483-94. doi: 10.1007/s00253-018-9022-5.
12. Lin JW, Guan SY, Duan ZW, Shen YH, Fan WL, Chen LJ, et al. Gene cloning of a novel fungal immunomodulatory protein from *Chroogomphis rutilus* and its expression in *Pichia pastoris*. *Journal of Chemical Technology & Biotechnology*. (2016) 91:2761-8. doi: 10.1002/jctb.4881.
13. Ko JL, Hsu CI, Lin RH, Kao CL, Lin JY. A new fungal immunomodulatory protein, fip-fve isolated from the edible mushroom, *Flammulina velutipes* and its complete amino acid sequence. *European Journal of Biochemistry*. (1995) 228:244-9. doi: 10.1111/j.1432-1033.1995.tb20256.x.
14. Wang Y, Gao YN, Bai R, Chen HY, Wu YY, Shang JJ, et al. Identification of a novel anti-cancer protein, fip-bbo, from *Botryobasidium botryosum* and protein structure analysis using molecular dynamic simulation. *Sci Rep*. (2019) 9:5818. doi: 10.1038/s41598-019-42104-1.
15. Li SY, Shi LJ, Ding Y, Nie Y, Tang XM. Identification and functional characterization of a novel fungal immunomodulatory protein from *Postia placenta*. *Food and Chemical Toxicology*. (2015) 78:64-70. doi: 10.1016/j.fct.2015.01.013.
16. Li SY, Jiang ZH, Sun LC, Liu X, Huang Y, Wang FZ, et al. Characterization of a new fungal immunomodulatory protein, fip-dsq2 from *Dichomitus squalens*. *Journal of Biotechnology*. (2017) 246:45-51. doi: 10.1016/j.jbiotec.2017.02.006.
